# A rare case of *Clostridium paraputrificum* bloodstream infection in a patient with intestinal necrosis: case report and literature review

**DOI:** 10.3389/fmed.2025.1702526

**Published:** 2025-12-03

**Authors:** Caixia Ji, Ke Wang, Zongyao Chen, E. Jianfei, Yayun Jiang, Ziqian Huang, Wei Huang

**Affiliations:** 1 Department of Clinical Laboratory, People′s Hospital of Deyang City, Deyang, Sichuan, China; 2 Pathogenic Microbiology and Clinical Immunology Key Laboratory of Deyang City, Deyang People's Hospital, Deyang, Sichuan, China; 3 Department of Clinical Laboratory, Yongchuan People′s Hospital of Chongqing, Chongqing, China; 4 Department of Blood Transfusion, People′s Hospital of Deyang City, Deyang, Sichuan, China; 5 Department of Pancreatitis Treatment Center, People′s Hospital of Deyang City, Deyang, Sichuan, China

**Keywords:** *Clostridium paraputrificum*, anaerobic bloodstream infections, necroticsmall bowel resection, case report, whole-genome sequencing, literature review

## Abstract

Bloodstream infections caused by anaerobic bacteria present a serious threat to patients. Rapid and accurate diagnosis is crucial for treatment and patient prognosis. Herein, we report a rare case of *Clostridium paraputrificum* bacteremia in an 82-year-old woman who developed an infection after undergoing necrotic small bowel resection. The patient was treated with 5-day anti-infective therapy with meropenem and linezolid, successfully controlling the disease. We also constructed a phylogenetic tree with other similar bacteria using gene sequencing and showed the virulence and antimicrobial resistance of *C. paraputrificum*. Additionally, the clinical features and antibiotic treatment of this case were reviewed and discussed in the existing literature. This case and review illustrate the insidious nature of *C. paraputrificum* infections, emphasizing the need for greater clinician awareness and improved diagnostic and treatment strategies.

## 1 Introduction

Bacteria can be classified according to their oxygen requirements into aerobes, anaerobes, and facultative anaerobes (1). Anaerobes are bacteria that thrive better under anaerobic conditions than in aerobic environments (2). *Clostridium* species are Gram-positive anaerobic bacilli, including 210 species and 5 subspecies. Clinically significant Clostridia species include *C. tetani*, *C. perfringens*, *C. botulinum,* and *Clostridioides difficile* (*C. difficile*) (3). Anaerobic bacteremia is a serious threat to the life and health of patients, with a mortality rate as high as 63% (4). If the mucosal barrier is compromised, it can enter the bloodstream and develop severe and potentially harmful infectious diseases. *C. paraputrificum* infections have now been reported in only 1% of all cases of *Clostridium* infections, and their clinical significance has not been fully described (5). Herein, we report an 82-year-old woman who developed *C. paraputrificum* bacteremia after undergoing a necrotizing small bowel resection for intestinal obstruction with necrosis. Timely antibiotic treatment effectively controlled the infection, gradually stabilizing the patient’s condition. This is likely the first reported case of bloodstream infection caused by *C. paraputrificum* in China.

## 2 Case presentation

An 82-year-old woman with a history of appendectomy was urgently admitted due to intermittent abdominal colic. On admission, vital signs were as follows: temperature, 36.5 °C; pulse, 84 beats/min; respiratory rate, 22 breaths/min; and blood pressure, 173/84 mmHg. Blood tests were unremarkable. Examination showed diffuse abdominal pain with pronounced rebound tenderness. CT indicated intra-abdominal hernia, intestinal ischemia, and obstruction in the right middle and lower abdomen, leading to the diagnosis of intestinal obstruction with necrosis. The following day, the patient underwent resection of the necrotic small intestine under general anesthesia.

Five days postoperatively, the patient developed chills that persisted for approximately 2 h, with her body temperature reaching 37.5 °C and peaking in the evening. A blood culture was performed immediately, and a repeat computed tomography scan revealed discontinuity in the anastomotic wall, accompanied by localized peritoneal fluid collection, confirming an anastomotic fistula. Given the persistent symptoms and radiological findings, she underwent a second laparotomy, during which the necrotic segment of the small bowel adjacent to the previous anastomosis was resected, and an ileal single-lumen fistula was created to divert intestinal contents.

**Figure 1:**
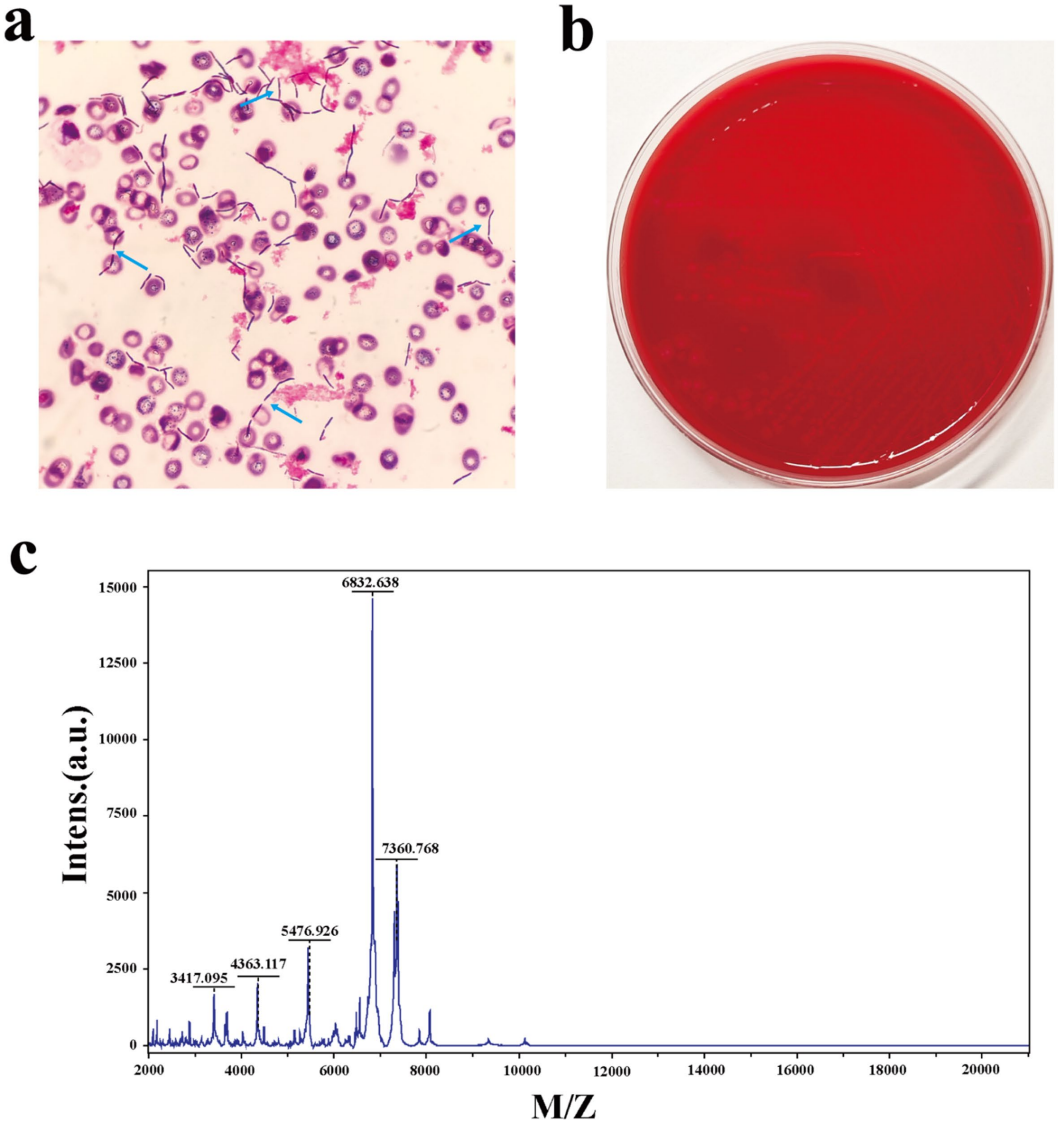
**(a)** Gram stain of a blood culture showing gram-positive rods (×100). Arrows show the bacteria under the microscope. **(b)** Colonies were seen on a blood agar plate after a 2-day anaerobic conditions culture transferred from the positive blood culture bottles (an anaerobic environment of 35 °C). **(c)** Matrix-assisted laser desorption and ionization time-of-flight mass spectra from the cultured colony.

**Figure 2:**
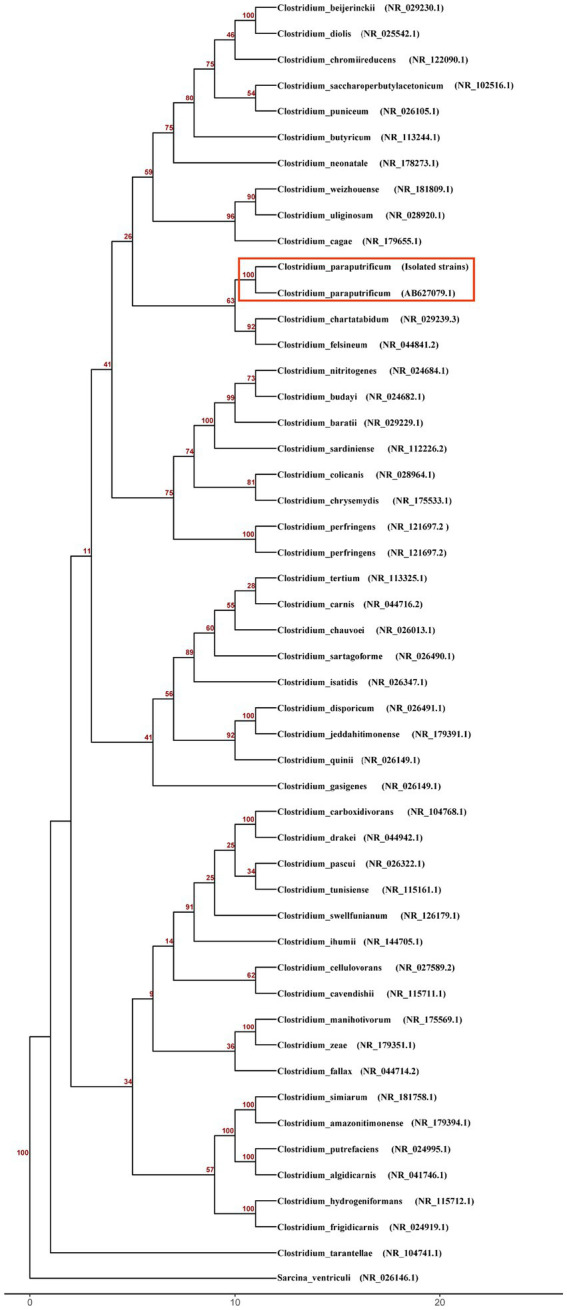
Phylogenetic analysis of Clostridium species based on 16S rRNA gene sequences. The neighbor-joining tree was constructed using the Kimura 2-parameter model and rooted with *Sarcina ventriculi* as the outgroup. Bootstrap values from 100 replicates are shown as decimal numbers at the nodes. Scale bar represents the number of nucleotide substitutions per site.

Three days after the second laparotomy, both anaerobic bottles of two sets of blood cultures turned positive. Gram staining showed Gram-positive rods (Figure 1a). Subculture on blood agar at 35 °C yielded no growth aerobically, but anaerobic incubation for 48 h produced grayish-white, irregular colonies with scalloped edges (Figure 1b). Matrix-assisted laser desorption/ionization time-of-flight mass spectrometry (Bruker LVD MALDI Biotyper) identified the bacterium as *C. paraputrificum* (score 2.07) (Figure 1c), with confirmation via whole-genome sequencing (Illumina, Inc., USA, Illumina NovaSeq 6,000) and 16S rRNA analysis (NCBI). Genomic analysis included phylogenetic tree construction using a custom R script, along with analysis of virulence-associated genes using the Virulence Factor Database (VFDB) (6) and identification of antimicrobial resistance (AMR) genes through the Comprehensive Antibiotic Resistance Database (CARD) and MEGARes database (7, 8). This integrated approach revealed multiple genes associated with both virulence and AMR (Figure 2; Table 1).

**Table 1:** Virulence analysis and antimicrobial resistance analysis of *Clostridium paraputrificum.*

Sequence	Start	End	Strand	Gene	Database	Accession
CP373A1_19	157,965	158,773	–	*hasB*	VFDB	WP_009880737.1
CP373A1_19	162,461	163,443	–	*cps4J*	VFDB	WP_000077424.1
CP373A1_26	352,996	353,396	–	*clpP*	VFDB	NP_465991
CP373A1_32	171,576	173,134	–	*clpC*	VFDB	NP_463763
CP373A1_28	77,979	78,481	–	*efrB*	CARD/MEGARes	WP_002289400.1
CP373A1_32	91,351	91,855	–	*efrB*	CARD/MEGARes	WP_002289400.1
CP373A1_28	79,694	80,522	–	*efrA*	MEGARes	WP_104671188.1

**Figure 3:**
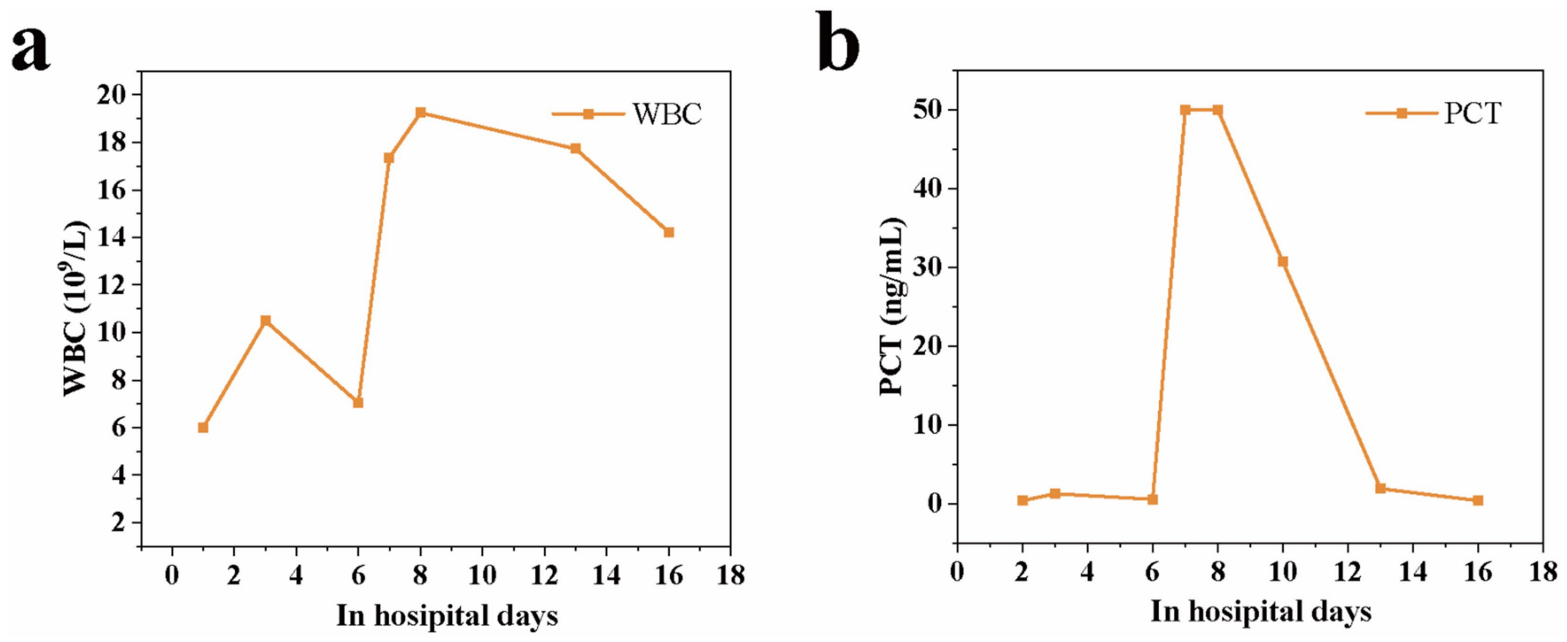
CRP and PCT in the blood throughout the treatment duration.

Antimicrobial susceptibility testing was performed based on the identified pathogen to guide targeted therapy. The Anaerobe ID&AST Test Card (Mindray Bio-Medical, China) showed *C. paraputrificum* was sensitive to ampicillin/sulbactam, piperacillin/tazobactam, cefoxitin, meropenem, metronidazole, chloromycetin, and vancomycin, but resistant to tetracycline and clindamycin. The patient was treated with meropenem (1 g every 8 h) and linezolid (0.6 g every 12 h). After 5 days of antibiotic treatment, the inflammatory markers—C-reactive protein (CRP) and procalcitonin (PCT)—returned to normal levels, indicating that the infection was under control (Figure 3). The patient was then transferred to a specialized department for further treatment and was discharged after 10 days when her condition stabilized.

## 3 Literature review and discussion

Anaerobic bacterial infections are diverse and common in abdominal, pelvic, complex skin, soft tissue, and bloodstream infections (2). In cases of intestinal necrosis or postintestinal resection, the anaerobic bacteria can translocate through damaged intestinal mucosa, with *C. paraputrificum* being particularly rare in such contexts. These infections usually involve mixed aerobic and anaerobic bacteria, where aerobes reduce environmental oxygen to facilitate anaerobe growth. Anaerobic cultures require specialized environments, and traditional identification methods are complex and time-consuming, making rare species such as *C. paraputrificum* easily overlooked (9). Additionally, anaerobes have complex genetic structures and act as potential reservoirs of AMR genes from other species (10).

*Clostridium* is a gram-positive, anaerobic or microaerophilic, coarse bacillus, causing diseases such as tetanus and gas gangrene (11). Clostridium bacteremia has a mortality rate of 29–35% (12). *C. paraputrificum* is rare, accounting for only 1% of reported Clostridium infections, and is even less frequently associated with intestinal necrosis or postabdominal surgery bloodstream infections compared to other Clostridium species.

To our knowledge, this is the first reported case of *C. paraputrificum* bloodstream infection in China, highlighting the infection′s complexity and insidious nature. Babenco et al. first reported a *C. paraputrificum* bacteremia case in 1976 involving an elderly man with myeloproliferative disease and necrotizing colon cancer who died from an infected aneurysm (13). Our review of 12 cases (Table 2) shows that these infections are linked to patient-specific factors, including advanced age (5, 13, 14), HIV infection (15, 16), digestive tract malignancies (13, 17), and gastrointestinal disorders (14, 18). These risk factors increase the likelihood of *C. paraputrificum* bloodstream infections by weakening immune function, enhancing inflammatory responses, or impairing mucosal barrier function. Impaired immune function leads to defective defense mechanisms, making it easier for pathogens to multiply and harder to clear, thereby significantly increasing the risk of bloodstream infections. Similarly, inflammatory mediators released by inflammatory responses will damage the vascular endothelium throughout the body and facilitate the invasion of pathogens. Age is also a significant risk factor. With aging, intestinal epithelial tight junction proteins may undergo remodeling, leading to increased colonic permeability. Increased intestinal permeability will make it easier for bacteria to pass through the intestinal wall and enter the bloodstream, causing bloodstream infections.

**Table 2:** Summary of the reported cases of *Clostridium paraputrificum* bloodstream infection.

Year	Age	Sex	Source of infection	Underlying disease	Treatment	Outcome	Ref
1976	88	Male	Necrotic colonic carcinoma	Polycythemia vera, adenocarcinoma in the colon	/	Died	(28)
1980	10	Female	/	Sickle cell disease	Penicillin	Cured	(29)
1982	65	Male	Aspiration Pneumonia	Hepatic disease	Penicillin	Cured	(30)
1988	32	Female	Necrotizing enterocolitis	Chronic non-cyclic neutropenia	Ampicillin	Cured	(31)
1996	32	Male	Obstructive duodenal	AIDS, Kaposi’s sarcoma	Metronidazole	Died	(16)
2015	65	Male	Colonic necrosis	AIDS	Vancomycin, Piperacillin/Tazobactam, Metronidazole	Cured	(15)
2017	88	Male	/	Hypertension, pyogenic spondylitis	Ampicillin/Sulbactam	Cured	(5)
2018	23	Female	Pyogenic liver abscesses	Hepatic adenoma	Vancomycin, Levofloxacin, Metronidazole	Cured	(32)
2020	78	Male	Colon neoplasm	Intestinal carcinoma, liver neoplastic	Metronidazole	Cured	(17)
2021	74	Male	Pseudomembranous colitis	Hypertension, iron deficiency, anemia	Vancomycin, Meropenem, Metronidazole	Died	(33)
2023	77	Male	Colonic pseudo-obstruction	Prostate cancer, hypertension, hyperlipidemia, hyperuricemia,	Ampicillin/Sulbactam	Cured	(18)
2023	59	Male	/	AIDS, HCV, HBV, diabetes, and hypertension	Meropenem, Vancomycin	Died	(34)

### 3.1 Gene of virulence and AMR

We analyzed the genome sequence of *C. paraputrificum* and identified several virulence and antibiotic resistance-related genes using the VFDB, CARD, and MEGARes databases. According to the VFDB, *C. paraputrificum* activates the virulence genes *hasB*, *cps4J*, *clpP*, and *clpC*. AMR analysis using CARD and MEGARes indicated the presence of *efrA* and *efrB* in *C. paraputrificum*. These genes are associated with bacterial virulence, stress response, and antibiotic resistance. The *hasB* is essential for hyaluronic acid capsule synthesis and contributes to virulence by facilitating immune evasion (19). The *cps4J* gene participates in capsular polysaccharide synthesis and immune modulation, thereby enhancing bacterial virulence and immune evasion (20). *ClpP* and *ClpC* function together as a proteolytic complex (ClpCP) that maintains protein homeostasis and regulates bacterial stress tolerance and virulence, making them important contributors to pathogenicity (21, 22). EfrAB is an ATP-dependent multidrug efflux pump that exports various antimicrobial agents, particularly fluoroquinolone antibiotics, thereby lowering their intracellular concentration and contributing to multidrug resistance (23, 24). Additionally, studies have shown that antibiotic-resistance genes in human intestinal bacteria can be exchanged within the microbiota and transferred to other bacteria (18). Such bacteria may serve as a potential reservoir for antibiotic resistance genes in other species.

### 3.2 Drug choice

Selecting antibiotics for anaerobic bacteria presents significant challenges. Research indicates that anaerobic infections are predominantly mixed infections (25). Therefore, it is crucial to remain vigilant with high-risk patients to avoid misdiagnosis. Moreover, increasing evidence shows that anaerobic bacteria are developing resistance to antibiotics, including high-grade options such as imipenem, piperacillin-tazobactam, ampicillin-sulbactam, and metronidazole (26). Suboptimal antibiotic treatment may select resistant bacteria and even induce shifts in resistance determinants. *C. paraputrificum* is rarely isolated clinically, and research on its antibiotic sensitivity is extremely limited. Therefore, selecting the appropriate antibiotic for patients diagnosed with *C. paraputrificum* infection is a major challenge. Inappropriate antibiotic use can lead to the emergence of drug-resistant bacteria and increase patient mortality (27). We reviewed all available literature on *C. paraputrificum* infections (Table 2). Empirical treatment should consider antibiotics such as vancomycin, metronidazole, and imipenem, either alone or in combination. Given the reported resistance to clindamycin, erythromycin, tetracycline, and penicillin, these should not be used as empirical treatments.

## 4 Conclusion

In conclusion, this case reports a *C. paraputrificum* bloodstream infection following necrotic small bowel resection, confirmed via anaerobic blood culture and mass spectrometry. Notably, the infection exhibits an insidious clinical profile, characterized by mild fever despite markedly elevated inflammatory markers, underscoring the need for vigilant monitoring of high-risk populations such as postintestinal necrosis patients and timely blood culture testing. Furthermore, genome sequencing clarified the isolate′s phylogenetic position and identified critical virulence and AMR genes, offering insights into its pathogenic mechanisms. Finally, both *in vitro* susceptibility data and literature evidence support vancomycin, metronidazole, or imipenem as monotherapy or in combination for effectively managing *C. paraputrificum* bloodstream infections.

## Data Availability

The datasets presented in this study can be found in online repositories. The names of the repository/repositories and accession number(s) can be found in the article/supplementary material.
