# Correction: A rare case of *Clostridium paraputrificum* bloodstream infection in a patient with intestinal necrosis: case report and literature review

**DOI:** 10.3389/fmed.2025.1761583

**Published:** 2026-01-06

**Authors:** Frontiers Production Office

**Affiliations:** Frontiers Media SA, Lausanne, Switzerland

**Keywords:** *Clostridium paraputrificum*, anaerobic bloodstream infections, necroticsmall bowel resection, case report, whole-genome sequencing, literature review

Authors Yayun Jiang and Ziqian Huang were erroneously not assigned as corresponding authors. The correct corresponding authors are:

Yayun Jiang, dyjiangyayun@163.com

Ziqian Huang, hzq-fy25@outlook.com

Wei Huang, 1337539781@qq.com

The original version of this article has been updated.

